# Efficacy of transcranial magnetic stimulation treatment in reducing neuropsychiatric symptomatology after traumatic brain injury

**DOI:** 10.3389/fneur.2024.1412304

**Published:** 2024-10-09

**Authors:** Gianna Carla Riccitelli, Riccardo Borgonovo, Mariasole Villa, Emanuele Pravatà, Alain Kaelin-Lang

**Affiliations:** ^1^Non-Invasive Brain Stimulation Research Unit, Neurocenter of Southern Switzerland, EOC, Lugano, Switzerland; ^2^Faculty of Biomedical Sciences, Università della Svizzera Italiana, Lugano, Switzerland; ^3^Neuroradiology Research Unit, Neurocenter of Southern Switzerland, EOC, Lugano, Switzerland; ^4^Department of Neurology, Inselspital, Bern University Hospital, Bern, Switzerland

**Keywords:** transcranial magnetic stimulation, traumatic brain injury, emotions, obsessive-compulsive disorder, neuropsychiatry, executive control

## Abstract

**Background:**

Neuropsychiatric disorders are highly disabling in traumatic brain injury (TBI) patients, and psychopharmacological treatments often fail to adequately mitigate their detrimental effects. Repetitive transcranial magnetic stimulation (rTMS) is an emerging treatment in neurology and psychiatry, showing potential in treating psychiatric disorders.

**Objective:**

This study investigates the efficacy of a novel, dual-site sequential rTMS protocol designed to treat neuropsychiatric symptoms in a TBI patient who was refractory to conventional treatments.

**Methods:**

A 34-year-old woman with severe head trauma and complex psychopathology underwent 20 daily sessions of focal-coil rTMS, combining inhibitory stimulation (1 Hz) on the right dorsolateral prefrontal cortex (DLPFC) and excitatory (10 Hz) on the left DLPFC, guided by a neuronavigation system. Psychiatric and neurocognitive assessments were conducted at baseline and at 2, 4, and 8 weeks following the beginning of rTMS treatment.

**Results:**

After 2 weeks of treatment, the patient showed decreased impulsivity and obsessive-compulsive symptoms, along with improvements in attention and processing speed. After 4 weeks, impulsivity further declined, though no other significant changes were noted. At 8 weeks, a persistent positive effect was observed, including enhanced positive emotions.

**Discussion:**

These findings suggest that guided, alternating neurostimulation of the DLPFC may modulate activity within cortico-striato-thalamo-cortical circuits, providing a promising alternative for managing neuropsychiatric symptoms in TBI patients who are resistant to traditional treatments.

## 1 Introduction

Neuropsychiatric disorders affect up to 88% of traumatic brain injury (TBI) survivors (1). The symptomatology of such brain damage manifests in various psychopathological conditions, including personality changes, impulsivity, severe irritability, affective instability, and delusions (2).

Pharmacotherapy and cognitive behavioral therapy are considered first-line treatments for TBI patients (1). However, approximately half of these patients are refractory to medical treatment and require augmentation strategies or advanced treatments (3). Repetitive transcranial magnetic stimulation (rTMS) is a non-invasive and outpatient therapy, which is gaining traction in the field of neurology and psychiatry (4).

Inhibitory rTMS protocols targeting the right prefrontal cortex (PFC) have shown efficacy in reducing obsessive-compulsive disorder (OCD) symptoms (5, 6) and depression post-TBI (7, 8). Moreover, excitatory rTMS protocols on the dorsolateral prefrontal cortex (DLPFC), bilaterally, have shown promise in managing a variety of psychiatric conditions, including depression (9), borderline personality disorders (BPD) (10, 11), and post-traumatic stress disorder (12). A recent study (13) compared two types of rTMS frequencies (inhibitory and excitatory) applied to the DLPFC, showing that both contribute to reducing impulsiveness, affective instability, and anger in patients with BPD.

Neuroimaging studies highlight the amygdala and PFC as two critical components of the brain’s circuitry that regulate personality and emotions (14, 15). Moreover, each amygdala has unique connections with different brain areas—the right amygdala with the contralateral area, basal ganglia, and frontal cortex, and the left amygdala with the anterior cingulate, right occipital, and left middle temporal gyrus. Disrupted connectivity in the amygdala is linked to various psychiatric disorders or populations who are at genetic risk for such illnesses (16, 17).

The PFC also plays a crucial role in regulating emotions throughout the brain (18). Damage to the right PFC can exacerbate negative emotions such as sadness or irritability, while damage to the left PFC can diminish positive emotions and motivation, which are closely associated with depression (19, 20).

Based on this evidence, the present study explores a dual-site rTMS treatment that combines inhibitory and excitatory stimulation on the DLPFC to modulate the frontolimbic network and improve symptoms in a TBI patient with complex psychopathology.

The primary outcomes measured were changes in impulsivity, OCD symptoms, and emotion regulation at 2, 4 and 8 weeks after the beginning of rTMS treatment, compared to baseline performance.

## 2 Materials and methods

### 2.1 Participant

The patient is a 34-year-old right-handed woman with 12 years of education, currently unemployed due to professional incapacity. At the age of 18, she met with a severe road traffic accident, resulting in polytrauma and a complex psychopathological condition. Prior to the accident, the patient was in good physical and psychological health.

The polytrauma led to a multifaceted clinical scenario, including penetrating injuries at the thoracic level, which caused a hypertensive pneumothorax, and at the abdominal level, leading to pneumoperitoneum. Additionally, the patient sustained severe contusions to both the head and the spine. The cranial trauma was particularly severe, inducing a coma due to bilateral subdural hematomas that required an emergency craniotomy. There was also widespread damage to the axons within the brain’s subcortical white matter.

**Figure 1:**
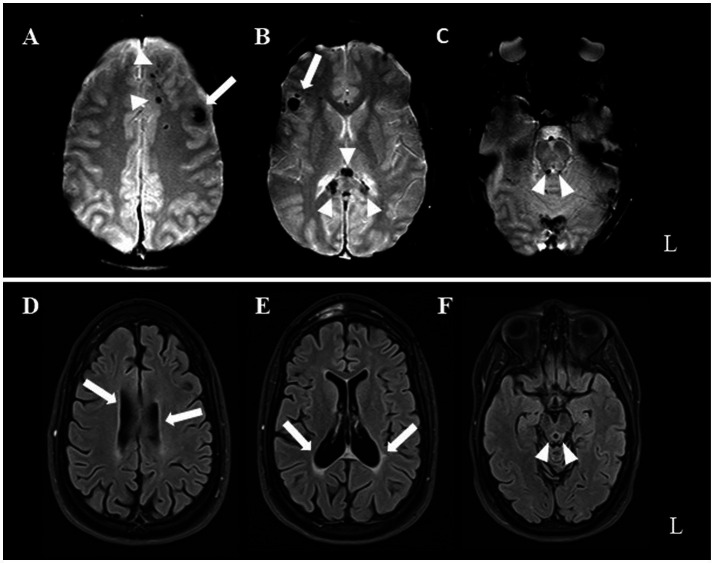
Upper row illustrates early post-injury axial T2*-weighted MRI. Hemorrhagic hypointense foci mark brain contusions at the level of the left forceps minor (arrowheads in A), left dorsolateral prefrontal cortex (arrow in A), right inferior frontal gyrus (arrow in B), splenium of corpus callosum (arrowheads in B), and periaqueductal mesencephalon tegmental region (arrrowheads in C). Lower row (D through F same acquisition planes as above) presents patient’s 13-year follow-up MRI. T2-FLAIR weighted images show occurrence of severe lateral ventricular (arrows) and aqueductal enlargement (arrowheads), related to parenchymal volume loss.

Post-recovery, the patient exhibited a range of neurological symptoms, including cerebellar ataxia and right pyramidal syndrome, alongside cognitive impairments (deficits in executive function and attention) and behavioral issues (frontal disinhibition). In January 2008, an MRI of the brain revealed hemosiderin deposits, which are indicative of axonal damage at the subcortical level, primarily in the frontotemporal and temporobasal regions (predominantly on the left), along the trunk of the corpus callosum, and in the right upper paravermial region (Figure 1; upper row).

In the years following the accident, the patient experienced a partial improvement in her neurological symptoms but continued to suffer from severe psychiatric issues. These issues included impulsivity that lead to destructive behavior and self-harm, delusions, mood instability with depressive episodes, emotional suppression, and obsessive-compulsive behaviors.

The severity of these symptoms often required emergency psychiatric interventions, including compulsory hospital admissions. The treatment involved a combination of antipsychotic medications (clotiapine, quetiapine, and clozapine) and mood stabilizers (valproic acid, oxcarbazepine, and lithium).

At the last clinical visit (June 2023), the patient’s neurological and psychiatric conditions remained stable, with no significant improvement. A follow-up brain MRI in February 2021 (T2-FLAIR; Scanner: 3 Tesla Skyra [Siemens, Erlangen, Germany]; TR = 8,000 ms, TE = 85 ms, FA = 150 deg., TI = 2,372 ms, FOV = 220*220 mm, matrix = 256*179, slice thickness = 3 mm, interslice gap = 0.3 mm; acquisition time = 3′42″) revealed minimal changes compared to the previous MRI (T2* gradient echo; scanner: 1.5 Tesla Skyra [Siemens, Erlangen, Germany]; TR = 800, TE = 31, FA = 35 deg., FOV = 230*200, matrix = 256*168, slice thickness = 5 mm, interslice gap = 0.8 mm, acquisition time = 2′13″). The MRI revealed slight further enlargement of the ventricles and aqueduct, indicating parenchymal volume loss (Figure 1; lower row).

At the time, the patient was on psychopharmacological treatment consisting of lithium (1,350 mg/day) and quetiapine (450 mg/day) in titration. Given her partial response to previous treatments and the persistence of disabling behavioral symptoms, rTMS treatment was proposed.

### 2.2 Neurological, psychiatric, and neurocognitive assessment

**Figure 2:**
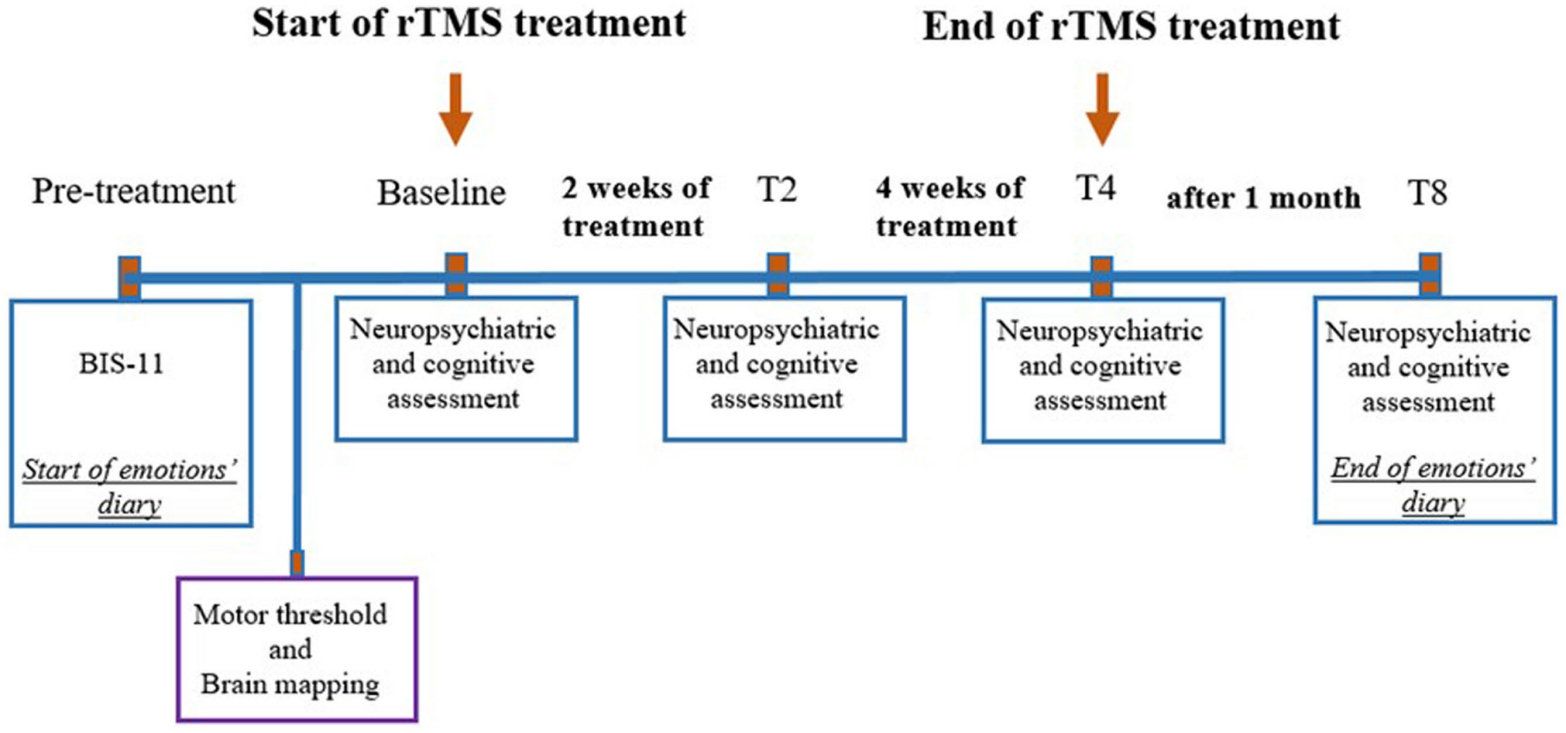
Timeline of events related to treatment. rTMS, Repetitive transcranial magnetic stimulation; BIS11, Barratt Impulsiveness Scale, T2, 2 weeks after the beginning of the treatment; T4, 4 weeks after the beginning of the treatment; T8, 8 weeks after the beginning of the treatment (follow-up).

The study timeline is illustrated in Figure 2.

The neurological examination included an assessment of movement disorders, possible side effects of medical therapy (iatrogenic), and psychiatric evaluation using the Clinical Global Impression Scale (CGI) (21). This scale was used to evaluate global illness severity (CGI-S), overall improvement from the start of treatment (CGI-I), and therapeutic response (CGI-E).

To assess impulsivity and obsessive-compulsive symptoms, the Barratt Impulsiveness Scale (BIS-11) (22) and the Yale-Brown Obsessive-Compulsive Scale (YBOCS) (23) were administered, respectively.

The neurocognitive evaluation tested sustained attention, cognitive flexibility, and processing speed using the Color Trails Test (24), and working memory and interference control using the Night and Day Test (25). The results from the neuropsychological tests were scored using a standardized method (26).

All assessments were conducted at four time points:

1. before rTMS treatment (baseline),
2. two weeks after the start of rTMS treatment,
3. four weeks after the start of rTMS treatment, and
4. eight weeks after the start of rTMS treatment (follow-up).

To control for social desirability bias, two pre-treatment BIS-11 measures were compared (one taken a week before treatment and one just before the treatment began).

In addition to the psychiatric and neurocognitive assessment, starting 1 week before the rTMS treatment, the patient was asked to maintain a diary to track daily mood fluctuations. The diary recorded both positive emotions (e.g., happiness, enjoyment, and satisfaction) and negative emotions (e.g., sadness, anger, fear, surprise, melancholy, loneliness, and annoyance), rated on a scale from 0 (“absent”) to 10 (“very high”).

### 2.3 Neuromodulation treatment

The rTMS protocol was delivered using a 70-mm cooled coil connected to a Magstim Rapid 2 stimulator (Magstim Co., Whitland, United Kingdom). On a separate day prior to the first treatment session, the resting motor threshold (RMT) for the right abductor pollicis brevis muscle was determined using an amplaid electromyograph (Fa. Micromed, Freiburg, Germany) according to the method of limits (27). The stimulation intensity for the experiment was set at 100% of the RMT.

A T1-weighted MRI scan (TR = 1,900 ms; TE = 2.1 ms; TI = 900 ms; FOV = 240 mm^2^; matrix = 256 × 256; voxel size = 0.9 × 0.9 × 0.9 mm^3^) of the patient was used as an anatomical reference. The target points, expressed in Talairach space, were automatically registered to the patient’s native space using SoftTaxic software.

To further enhance the accuracy of stimulation, we utilized an open-source software tool (SimNIBS version 3.2) (28) to position the TMS coil precisely where the electric field strength was optimal.

Briefly, the patient’s T1-weighted MRI data were processed in SimNIBS to create a personalized head model and simulate the electric field distribution from the TMS coil. The resulting 3D map of the electric field was exported and aligned with SofTaxic’s coordinate system, which then guided the TMS coil in real time, optimizing the stimulation by targeting the areas of maximum electric field strength. For additional information, please refer to the Supplementary materials (29, 30).

Within the course of 1 month, the patient underwent 20 daily rTMS sessions, with one session per day, 5 days a week. The treatment included both inhibitory and excitatory rTMS stimulation.

Inhibitory rTMS (1 Hz) was applied to the right DLPFC in a continuous 15-min train, delivering one pulse per second for a total of 900 pulses per session. Excitatory rTMS (10 Hz) was applied to the left DLPFC in 30 trains of 10 s each, with a 10-s interval between trains, for a total of 1,500 pulses per session. Each session began with stimulation of the right DLPFC, followed by the left DLPFC. The patient had no prior experience with rTMS before the study.

### 2.4 Statistical analysis

Given the exploratory nature of the study, clinical, neuropsychiatric, and cognitive changes were assessed using univariate descriptive statistics.

## 3 Results

The results of the psychiatric and neurocognitive assessments are reported in Table 1.

**Table 1:** Neuropsychiatric and cognitive assessment results over time.

Neuropsychiatric/cognitive test	Scores				Percentage change**		
	Baseline	T2	T4	T8	Baseline vs T2	Baseline vs T4	Baseline vs T8
BIS-11	70.5*	66	58	58	−6.4	−17.7	−17.7
BIS-11 Attentional impulsiveness	19*	17	14	16	−10.5	**−26.3**	−15.8
BIS-11 Motor impulsiveness	20*	18	17	16	−10	−15	−20
BIS-11 Non-planning impulsiveness	31.5*	31	27	26	−1.6	−14.3	−17.5
YBOCS	21	10	11	12	**−52.4**	−47.6	**−42.8**
YBOCS Obsessions	9	9	7	8	0	**−22.2**	−11.1
YBOCS Compulsions	12	1	4	4	**−91.7**	**−66.7**	**−66.7**
Night & day 1 Test (sec)	40.2	32.7	38	39	−18.7	−5.5	−2.9
Number of errors	0	0	0	0	–	–	–
Night & day 2 Test (sec)	57	58	64	63	1.7	12.3	10.5
Number of errors	0	0	0	0	–	–	–
Night & day 3 Test (sec)	119	118	103	128	−0.8	−13.4	7.5
Number of errors	21	6	5	7	**−71.4**	**−76.2**	**−66.7**
Color Trials Test 1 (sec)	100	110	98	90	10	−2	−10
Color Trials Test 2 (sec)	286	187	217	180	**−34.6**	**−24.1**	**−37.1**

BIS-11, Barratt Impulsiveness Scale; YBOCS, Yale Brown Obsessive-Compulsive Scale; T2, 2 weeks after the beginning of the treatment; T4, 4 weeks after the beginning of the treatment; T8, 8 weeks after the beginning of the treatment (follow-up). In bold positive changes greater than 20%. *mean of two scores taken 7 days before the beginning of rTMS treatment and at baseline **negative percentages represent improvements in performance.

At baseline, the extent of the patient’s illness was classified as severe (CGI = 6). Personality and behavior exhibited moderate disturbances (Barratt Impulsiveness Scale = 70.5; Yale-Brown Obsessive-Compulsive Scale = 21), while attentive and executive functions were impaired, displaying a significant number of errors due to diminished inhibition control.

After 2 weeks of treatment, notable improvements were observed in physical control, including reduced tremors, better posture, and improved fluidity in oral and gestural communication. Concurrently, there was a slight decrease in impulsivity (6.4%) and a significant reduction in obsessive-compulsive behavior (52.4%), with a substantial improvement in compulsive attitude (91.7%). Improvements were also observed in attentive-executive functions, including processing speed (18.7%), self-shifting (0.8%), and inhibitory control (34.6%), which led to a 71.4% increase in answer accuracy.

After 4 weeks, the patient experienced a reduction in illness severity to a moderate level (CGI = 4). Impulsivity control improved further, with a 17.7% reduction, and attentive impulsiveness showed a notable improvement of 26.3%. Although obsessive-compulsive tendencies increased slightly by 4.8%, the overall reduction remained significant at 47.6%. Cognitive performance in areas such as processing speed and sustained attention remained stable, while improvements in set-shifting and inhibitory control led to a further increase in answer accuracy, reaching 76.2%.

At the follow-up assessment, the neuropsychiatric and cognitive improvements observed earlier were still present, although there was a slight decrease in inhibitory control (13%) and response accuracy (10%).

**Figure 3:**
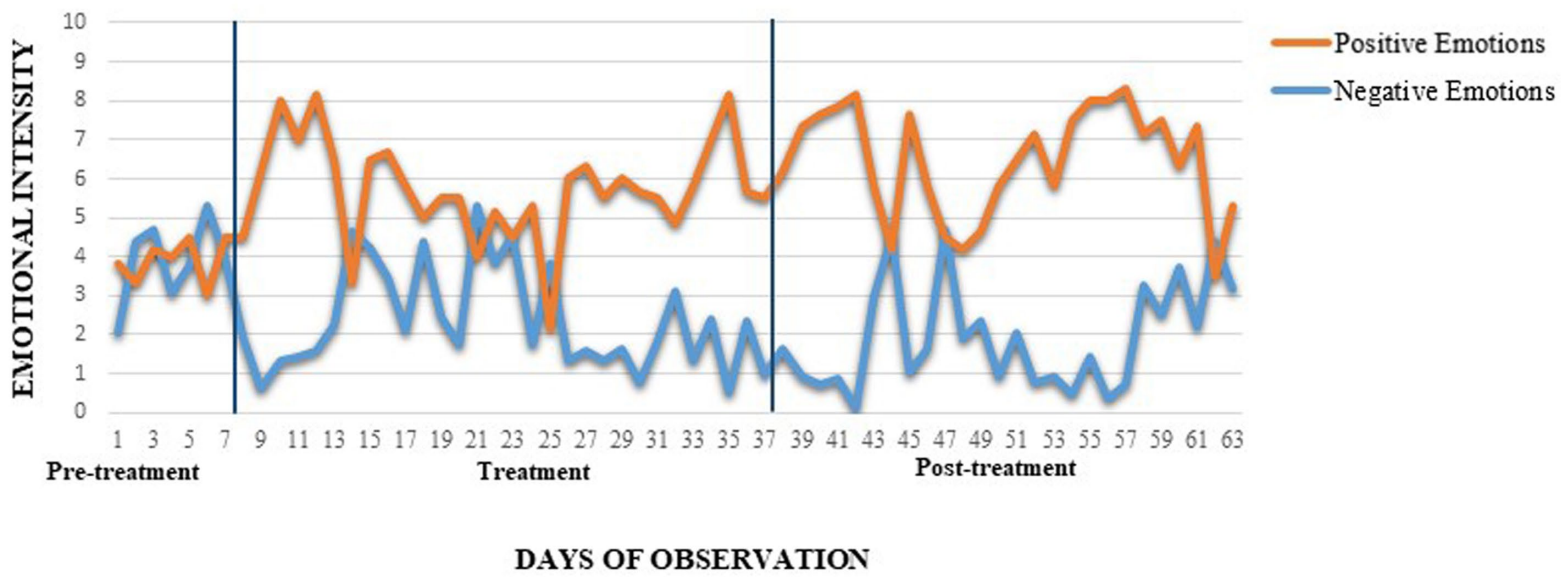
Daily self-report of positive and negative emotions intensity. BIS-11, Barratt Impulsiveness Scale; YBOCS, Yale Brown Obsessive-Compulsive Scale; T2, 2 weeks after the beginning of the treatment; T4, 4 weeks after the beginning of the treatment; T8, 8 weeks after the beginning of the treatment (follow-up). The two lines delimit treatment administration.

A qualitative increase in positive daily emotions was observed over time, with baseline mean = 3.9, rising to 5.9 after 2 weeks, 5.4 after 4 weeks, and 6.5 after 8 weeks. Simultaneously, there was a consistent decrease in negative emotions, from a baseline mean of 3.9 to 2.8 after 2 weeks, 2.2 after 4 weeks, and 1.9 after 8 weeks (Figure 3). These trends suggests a sustained improvement in emotional regulation over the course of the study.

## 4 Discussion

To the best of our knowledge, this is the first case to examine the effects of dual-site sequential focal coil rTMS, using 1 Hz (inhibitory) stimulation on the right DLFFC and 10 Hz (excitatory) stimulation on the left DLPFC, in a TBI patient with severe personality and emotional disorders.

Daily, sequential inhibitory and excitatory monitored stimulation was conducted to (1) increase the neuronal activity in the right subcortical prefrontal circuit to manage negative emotions (31) and obsessive-compulsive behavior (32); (2) stimulate the inhibitor control of the left DLPFC over the ipsilateral amygdala to regulate positive emotions (33, 34); and (3) increase interhemispheric connectivity to intensify the synaptic response of the basolateral amygdalae, thereby regulating amygdala-dependent behaviors (35).

The results indicate significant and lasting improvements in neuropsychiatric symptoms, with marked enhancements in clinical stability and social interactions.

Within 2 weeks, rTMS treatment led to improvements in physical balance, control, and posture, along with a reduction in obsessive-compulsive symptoms, executive dysfunction, and emotional instability.

Interestingly, previous findings have shown that inhibitory rTMS applied to the right DLPFC has a medium-term effect in reducing obsessive-compulsive symptoms and anxiety (5), suggesting that it rebalances prefrontal cortex activity. This rebalancing likely enhances executive function and control over impulsive, obsessive, and compulsive behaviors by modulating the activity of the cortico-striato-thalamo-cortical circuit (36).

After 4 weeks, rTMS treatment had a notable effect on impulsivity, particularly in reducing attentive impulsiveness, resulting in a substantial improvement in answer accuracy during cognitive tasks.

The treatment’s delayed impact on impulsivity dysregulation may be attributed to the complex nature of impulsivity, as its behavioral effects are often delayed due to learning processes that foster adaptive behaviors (37).

In line with previous findings on various psychiatric and personality disorders (10, 38, 39), we observed a qualitative increase in positive emotions (upregulation) and a decrease in negative emotions (downregulation) during treatment, with these effects persisting after 8 weeks. These emotional regulation improvements could be due to the sequential right and left rTMS stimulation, which contributes to emotional balance (40).

Finally, the sustained benefits of rTMS may be indicative of induced changes in cortical and subcortical synaptic efficacy and connectivity within the network responsible for controlling impulsivity, emotional instability, and emotional regulation.

The main limitation of this exploratory study is the absence of a control group. However, we are confident that the rigorous application of the rTMS intervention and the longitudinal assessment of within-subject changes provide valuable preliminary findings, serving as a reference point for future randomized control trials.

Additionally, our study did not assess motor threshold (MT) during TMS treatment, despite recent studies suggesting its potential to predict changes in symptomatology. Moreover, the application of neuroimaging techniques such as functional MRI to assess brain activity within the targeted network and quantitative electroencephalograms (EEG) for detailed analysis of electrical patterns could significantly enhance our understanding of the underlying mechanisms and the efficacy of rTMS in treating neuropsychiatric disturbances in TBI patients who are unresponsive to conventional medical treatments.

The current study provides preliminary evidence supporting the effectiveness of a sequential, alternated-frequency rTMS protocol in reducing impulsivity, OCD symptoms, and executive dysfunction in TBI patients. This preliminary evidence suggests that this rTMS protocol may have potential applications in the treatment of other conditions with similar symptom profiles, such as attention-deficit hyperactivity disorders (ADHD), Tourette’s syndrome, BPD, bipolar disorder, autism spectrum disorder, and OCD.

## Data Availability

The raw data supporting the conclusions of this article will be made available by the authors without undue reservation.
