# Supplementary material for: Efficacy of transcranial magnetic stimulation treatment in reducing neuropsychiatric symptomatology after traumatic brain injury
Source: Front Neurol. 2024 Oct 9;15:1412304. doi: 10.3389/fneur.2024.1412304 (PMC11496968; doi:10.3389/fneur.2024.1412304)
Supplement: Supplementary file 1 [file Data_Sheet_1.PDF]

Supplementary Material

Material and Methods: Neuromodulation treatment

The patient's T1-weighted MRI data previously acquired was input into SimNIBS (Simulation of Non-invasive Brain Stimulation) to generate a patient-specific finite element head model. After creating the head model, the TMS coil's position, orientation, and stimulation parameters were set within SimNIBS to simulate the electric field distribution within the brain. The result was a 3D map of the electric field, illustrating the areas of maximum and minimum stimulation.

Subsequently, the electric field simulation data, including the 3D coordinates of the field distribution and field strength, were exported from SimNIBS.

This exported data was then mapped onto the coordinate system used by SofTaxic. The mapping process involved aligning the SimNIBS-generated electric field data with the anatomical landmarks recognized by SofTaxic. The data was automatically converted into a format compatible with SofTaxic.

Once the data was imported into SofTaxic, it displayed the electric field distribution within the brain in real-time alongside the anatomical images. With the SimNIBS data integrated, SofTaxic was used to navigate the TMS coil to the precise location where the electric field strength was optimal, ensuring more effective and targeted stimulation.
